# Thirty-Year-Old Paradigm about Unpalatable Perch Egg Strands Disclaimed by the Freshwater Top-Predator, the European Catfish (*Silurus glanis*)

**DOI:** 10.1371/journal.pone.0169000

**Published:** 2017-01-06

**Authors:** Lukáš Vejřík, Ivana Vejříková, Luboš Kočvara, Zuzana Sajdlová, Son Chung Hoang The, Marek Šmejkal, Jiří Peterka, Martin Čech

**Affiliations:** 1 Biology Centre of the Czech Academy of Sciences, Institute of Hydrobiology, Na Sádkách 7, České Budějovice, Czech Republic; 2 Faculty of Science, University of South Bohemia in České Budějovice, Branišovská 31, České Budějovice, Czech Republic; University of Ostrava, CZECH REPUBLIC

## Abstract

So far, perch egg strands have been considered unpalatable biological material. However, we repeatedly found egg strands of European perch (*Perca fluviatilis*) in the diet of European catfish (*Silurus glanis*) caught by longlines in Milada and Most Lakes, Czech Republic. The finding proves that perch egg strands compose a standard food source for this large freshwater predatory fish. It extends the present knowledge on catfish foraging plasticity, showing it as an even more opportunistic feeder. Utilization of perch egg strands broadens the catfish diet niche width and represents an advantage against other fish predators. Comparison of datasets from extensive gillnet and SCUBA diver sampling campaigns gave the evidence that at least in localities where food sources are limited, multilevel predation by catfish may have an important impact on the perch population.

## Introduction

A study published 31 years ago by Newsome and Tompkins [1] described perch (*Perca* spp.) egg strands as repellent matter for predators. Observation and testing of six fish and four invertebrate species proved that perch egg strands are an undesirable food source for them. One year later, Diamond and Wakefield [2] published a topical study referring to the utilization of perch egg strands by three species of caddisfly larvae (Trichoptera) and two species of flatworms (Tricladida). The authors pointed out that these represent only a small fraction of aquatic invertebrates and predation pressure exerted on perch is irrelevant. The paradigm of perch egg strands as unpalatable biological matter has since been widely accepted by researchers all over the world [3–9].

European catfish (*Silurus glanis*), one of the world biggest freshwater fish, is a typical opportunist with a wide diet niche. It has successfully spread worldwide accompanied by human activity [10–14]. Recent studies demonstrated that European catfish uses atypical food sources including sources not originating from freshwater but marine or terrestrial ecosystems [15,16].

Although the European catfish is a top predator, only a few studies regarding its diet have been carried out, and most of them are recent (for review see [12]). The main reason is the poor capture success by standard ichthyological methods [17]. In the present study, we prove that perch (*Perca fluviatilis*) egg strands are a part of the European catfish diet. Further, we discuss the potential impact of European catfish on perch populations as a result of multilevel predation.

## Materials and Methods

### Study site

**Fig 1:**
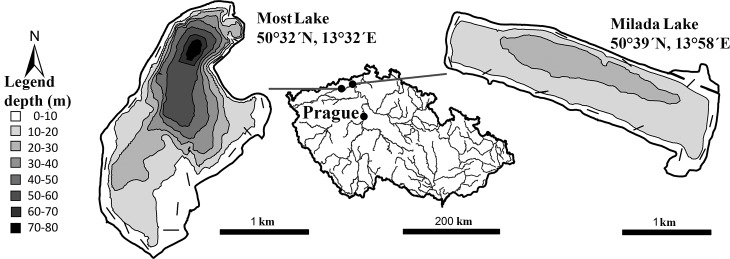
Map showing the location and relevant depths of the two study sites, Milada and Most Lakes, Czech Republic. Localities sampled by longlines are shown by lines along the lake shores.

The study was conducted in two water bodies created after aquatic restorations of mining pits, Milada and Most Lakes, Czech Republic. The oligo- to mesotrophic Milada Lake has an area of 250 ha, volume of 36×10^6^ m^3^ and maximum depth of 25 m (Fig 1). Aquatic restoration lasted from 2001 to 2011. Northern pike (*Esox lucius*) was introduced in 2005 (789 individuals, mean weight 0.3 kg) and European catfish in 2007 (316 individuals, mean weight 1.2 kg), both for biomanipulation purposes. The oligotrophic Most Lake has an area of 310 ha, volume of 70×10^6^ m^3^ and maximum depth of 75 m (Fig 1). Aquatic restoration lasted from 2008 to 2014. Northern pike (2332 individuals, mean weight 1.1 kg) and European catfish (694 individuals, mean weight 3.7 kg) were both introduced in 2011, 2012 and 2013. In both lakes, all catfish individuals were individually tagged with a passive integrated transponder tag (PIT-tag, Oregon RFID, full-duplex, length 12 mm, diameter 2.15 mm, weight 0.11 g, 11784/11785 compatible).

### Fish sampling and stomach content analysis

**Fig 2:**
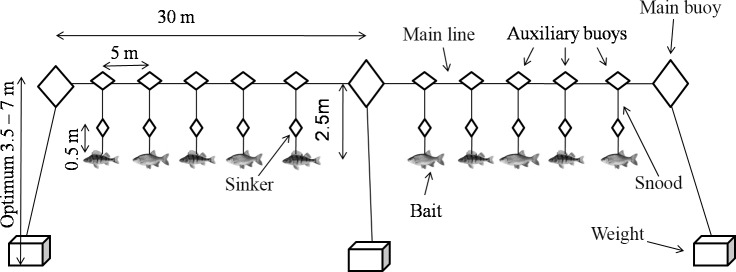
The scheme of longlines, fishing method used for catfish sampling in Milada and Most Lakes.

European catfish and northern pike from both lakes were caught by longlines in early May 2014 and 2015 during a 4-day-and-night-long campaign (see Fig 2 for illustrative description). Animal treatment (including method of longlines and stomach content analysis) was performed in accordance with the guidelines from the Experimental Animal Welfare Commission under the Ministry of Agriculture of the Czech Republic (Ref. No. CZ 01679) and with permission of Palivový kombinát Ústí, státní podnik, owner of the study sites. The work was approved by the Ethics Committee of the Czech Academy of Sciences. The field study did not involve endangered or protected species. The main line was 60 m long with three main buoys situated at both ends and in the middle of the line. Anchoring ropes, 3.5–7 m long, with weights (32 kg each) fixing the main line in place were tied to the buoys. Auxiliary buoys were situated every 5 m between the main buoys with a hanging 2.5 m long snood made of two parts i) 2-m long fishing-line with maximum load of 50 kg and ii) more durable 0.5 m long fishing-line with maximum load of 100 kg with a swivel between the two parts to preventing twisting. There was also a 150 g sinker keeping the snood at the appropriate depth. At the end of the snood, there was a catfish rig composed of a single hook with bait (total length of the bait: L_T_ = 180–300 mm) and a fishing treble. The predator (catfish or pike) was hooked by the fishing treble hanging under the baited hook while it tried to catch and tear down the bait. Altogether 30 individual bait fish on 3 longlines were used each day of sampling. To cover the shore area of the lake evenly, the lines were moved each day to a new place and they were checked three times per day (before dusk, soon after midnight, and shortly after dawn). Most of the catfish were caught during the night, most of the pike during the day. All individuals were measured, weighed and non-invasive stomach content analyses were provided. In the case of catfish, stomach content was extracted by hand through the opened mouth and gullet [18]. In the case of pike, water was pumped through a small tube into the pike´s stomach and the content was washed out through a larger tube into a jar [19]. The fish were released back into the lake as soon as possible. The stomach contents were consequently identified, or fixed by 70% ethanol in case of highly digested matter requiring precise laboratory identification using diagnostic elements including fish bones [20,21].

To obtain quantitative assessment of perch egg strands, SCUBA divers monitored their occurrence, numbers and sizes at transects from the shore to a depth of ~20 m in April and May in 2015 (for details of the methodology and results from previous years see [9, 22–25]).

Sizes of female perch that had contributed to the spawning were estimated from the width of individual egg strands, using the equation of Dubois et al. [26]. Perch abundance was estimated by multi-mesh gillnets (see [24] for detailed description). The gillnets were set overnight (September; installed 2 h before sunset, lifted 2 h after sunrise) at depths of 0–3, 3–6, 6–9 and 9–12 m in benthic and 0–5 and 5–10 m (or 0–6 and 6–12 m in 2014, respectively) in pelagic habitats at three to four localities. Altogether, 224 benthic and 74 pelagic gillnets were set, representing a total exposed area of 25,860 m^2^. Only 6% and 2% of fish community was annually caught by gillnets in Milada and Most Lakes, respectively. In case of perch population, it was 9% and 2% in Milada and Most Lakes, respectively (for more details see to supplementary materials).

### Abiotic factors

Water temperature measurements were taken between 12:00 and 14:00 during the sampling campaign in both years (2014 and 2015) and lakes. Measurements were made at 1 m depth intervals using a calibrated YSI 556 MPS probe (YSI Incorporated—Yellow Springs, Ohio, USA). Temperature of epilimnion was used for purpose of this study.

### Statistical analysis

To evaluate whether the trend in decreasing numbers of spawned egg strands was caused by decreasing size of the perch spawning stock, only perch ≥ 230 mm L_T_ were used in the analysis. This size limit was set for two main reasons: 1) The SCUBA diving results from 2007 and 2009 have shown that in Milada Lake 97% and 92% of perch egg strands were spawned by females ≥ 230 mm L_T_ [9, 23]. 2) Based on gillnet catches, almost 90% of perch ≥ 230 mm L_T_ were females [24]. Regression analysis was used for determining the relationship between CPUE (catch per unit effort) of perch ≥230 mm L_T_ caught by 1,000 m^2^ of gillnets in September of year X-1 and the CPUE of egg strands found by SCUBA divers (egg strands per 10 hours of diving) during spawning season in following spring, *i*.*e*. April/May of year X. The CPUE of perch caught by gillnets was calculated as the mean of catches of perch individuals ≥230 mm L_T_ over all depth layers of benthic and pelagic habitats. This comparison is not biased by the growing season because female perch of 230 mm L_T_ caught in September of year X-1 will be of approximately the same size in April/May of year X (for more details see [24]). The statistical test was performed in the R environment for statistical computing (version 3.2.2) [27]. A nonparametric Kruskal–Wallis test (Statistica 12; Stat-Soft Inc., Tulsa, OK) was used to test for differences between sizes of catfish with perch egg strands and catfish with other food items in the stomachs.

## Results

In total, 128 individuals of the European catfish (including three recaptures) were caught in Milada and Most Lakes during spring sampling in 2014 and 2015 (L_T_ range 710–1580 mm, weight range 0.8–23.5 kg). Altogether, 56 food items in the stomachs of 37 individuals were found. Perch egg strands were recorded in ten different catfish (verified by PIT-tags; one egg strand per catfish; Table 1). One of them had perch egg strand and juvenile perch in the stomach. Otherwise, only perch egg strands were found. In contrast, no perch egg strands were found in pike (41 individuals, L_T_ 690–1190 mm, weight 3.6–14.2 kg).Perch egg strands were not detected in stomachs of catfish in Milada Lake, in 2014, but in 2015. Excluding Milada Lake 2014, perch egg strands composed 13–71% of identified diet items (by numbers; Table 1). Catfish with perch egg strands in the stomachs were significantly smaller than catfish with other food items in the stomachs in both lakes (F_1,28_ = 5.6, *p* < 0.05; Table 1). All perch egg strands extracted from catfish stomachs included a jelly coat. The width of egg strands from catfish stomachs in Most Lake was 63 ± 8 mm (mean ± SD) in both years, corresponding with L_T_ of perch female 278 ± 20 mm. The width of egg strands from catfish stomachs in Milada Lake in 2015 was 50 and 55 mm, corresponding with L_T_ of perch female 242 and 256 mm.

**Table 1:** Total number of catfish, number of catfish with egg strands, with other food items and with empty stomach caught by longlines in Milada and Most Lakes in spring 2014 and 2015. Values in lines show number of catfish, size of catfish (mean L_T_ ±SD, in mm) and number of given stomach content (some individuals had more than one food items in the stomach). In Milada 2015, one individual is included in two categories (with egg strands, with other food items) because an egg strand and also a small perch were found in the stomach.

Lake		Most		Milada	
Year		2014	2015	2014	2015
Date of sampling		6.–9. 5.	6.–7. 5.	12.–15. 5.	5.–7. 5.
Water temp. °C		12. 1	12. 4	16. 3	12. 2
Number of catfish	Total	56 (850±161)	29 (877 ±122)	26 (1125±168)	17 (1153±215)
	With egg strands	3 (803±32), 3	5 (825±38), 5	0	2 (1070±113), 2
	With other food items	11 (915±102), 19 ^ǂ^	2 (898±96), 2 *	10 (1226±81), 17 ^&^	5 (1152±152), 8 ^#^
	With empty stomach	42 (836±175)	22 (887±134)	16 (1065±179)	11 (1196±261)

ǂ 8× rudd (*Scardinius erythrophthalmus*), 4× ruffe (*Gymnocephalus cernua*), 3× tench (*Tinca tinca*), 1× perch, 2× bird (Aves), 1× frog (Anura).

* 2× roach (*Rutilus rutilus*).

^&^ 9× rudd, 3× perch, 2× asp (*Aspius aspius*), 1× tench, 2× bird.

^**#**^ 7× perch, 1× rudd.

In Most Lake 2015, SCUBA divers found 3 perch egg strands of width >40 mm, corresponding with L_T_ of perch female >230 mm, during three dives in three consecutive weeks (total duration 3.5 h). In Milada Lake 2015, 23 perch egg strands were found during six dives in three consecutive weeks (total duration 9 h). The width of 11 of them was 30–40 mm, corresponding with L_T_ of perch female 180–213 mm, and 12 of them were wider than 40 mm, corresponding with L_T_ of perch female >230 mm.

**Fig 3:**
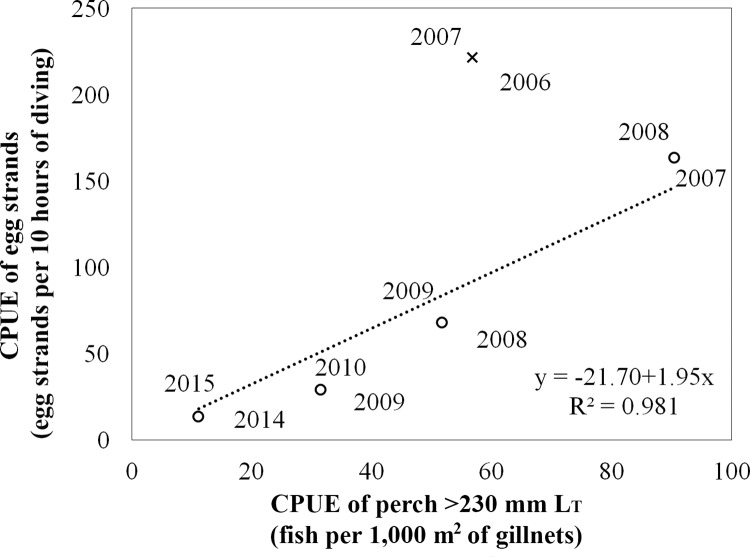
The relationship between the CPUE of perch ≥230 mm *L*_*T*_ caught by gillnets during late summer in Milada Lake (years 2006–2009, 2014) and the CPUE of perch egg strands found by SCUBA divers during the following spring (years 2007–2010, 2015). Regression analysis was provided for all years of monitoring (marked by empty circles), excluding the year marked by a cross (*i*.*e*. CPUE of perch ≥230 mm *L*_*T*_ caught by gillnets in 2006, and CPUE of perch egg strands found by SCUBA divers in 2007). The cross corresponds with the period before catfish mass stocking in Milada Lake, *i*.*e*. before impact of catfish on perch population.

According to gillnet sampling, an increase in perch abundance (L_T_ ≥ 230 mm) in Milada Lake was observed only between the years 2006 and 2007. A decrease in perch abundance has been observed since 2007, the year when catfish were stocked. A similar trend has been observed by SCUBA divers during perch egg strand monitoring. A strong relationship was found between the CPUE of perch ≥230 mm L_T_ caught by gillnets and the CPUE of perch egg strands found by SCUBA divers during the following springs (regression analysis; F_1,2_ = 179.2, *p* < 0.001; y = 4.27x-25.51, R^2^ = 0.98; Fig 3). The relationship was not observed between the perch abundance in 2006 and the number of perch egg strands in 2007, *i*.*e*. the period before catfish mass stocking in Milada Lake. At that time, one larger perch individual caught into the gillnets in late summer corresponded with three times more perch egg strands deposited next spring compared to following years (Fig 3; for complete dataset see S1 Data).

## Discussion

It is clear that perch egg strands were eaten after they were spawned because the occurrence of jelly coat indicates their deposition in water [1]. No fish remains were found along with perch egg strands in the stomachs, indicating that egg strands were consumed intentionally, not with female perch preyed on before spawning or during the spawning event. Only in one exception, we found a well-preserved perch (150 mm L_T_) along with a perch egg strand, but the strand width of 55 mm corresponds with perch female of 256 mm L_T_ [26] implying no relation. Catfish apparently eat perch egg strands directly from the flooded vegetation, which are frequently found in post-mining lakes and are commonly used by perch as spawning substrate [9, 22,24]. In one case, a small part of the spawning substrate (branch of a tree) was also found in the egg cluster extracted from the catfish stomach.

Perch egg strands were present in catfish stomachs in the case of Most Lake 2014 and 2015 and also Milada Lake 2015. Catfish most likely utilized perch egg strands also in Milada Lake in 2014, but water temperature of 16.3°C during longline sampling was already much higher than the optimal temperature for perch spawning, *i*.*e*. 12°C [28, 29, 24] and, most probably, the perch had probably already hatched. Unfortunately, direct monitoring of perch egg strands was not carried out in Milada Lake in 2014. Population of adult perch in Most Lake is three times higher in comparison to Milada Lake (28.4 vs. 8.6 ind. ≥230 mm L_T_ per 1,000 m^2^ of gillnets; [30,31]). Nevertheless the catfish population is also more abundant in Most Lake (1.86 ind. ha^-1^, 7.65 kg ha^-1^, mean L_T_ 850 mm) than in Milada Lake (0.72 ind. ha^-1^, 5.94 kg ha^-1^ mean L_T_ 1030 mm; Vejřík et al., in prep.). The catfish population represents 24.4% and 39.3% of the total fish biomass in Milada and Most Lakes, respectively. In Milada Lake, the populations of the top predator (only) is close to the average biomass of all predators (including e.g. perch ≥15 cm standard length) calculated for other aquatic ecosystems of a similar size in the Czech Republic [32], in Most Lake the biomass of the top predator almost doubled this value.

The low number of perch egg strands found in Most Lake in 2015 (3 pieces, *i*.*e*. 8.6 pcs. per 10 hours of diving) was probably influenced by high predation pressure by the smaller catfish. It is supported by the fact that catfish with perch egg strands in the stomachs were significantly smaller (mean 867 mm L_T_) than catfish with other food items in the stomachs (1067 mm L_T_). Smaller individuals are gape limited and their prey capture efficiency is lower, therefore readily available food sources such as egg strands are preferred. In Most Lake in 2015, catfish presence in perch spawning areas was also confirmed by SCUBA divers, where nine catfish were observed (per 3.5 h of diving) giving the ratio of three catfish to one perch egg strand.

Perch reproduction in Milada Lake has been intensively monitored for some years [9,25], previous years have shown an apparent decrease in the abundance of adult perch and a related decrease of perch egg strands since predators, catfish and pike, were stocked [24]. An increase in perch abundance was observed only between the years 2006 and 2007. Since 2007, the year of catfish mass stocking, a continuous decrease in the abundance of adult perch and subsequent decrease in abundance of perch egg strands has been observed. Čech et al. [24], monitoring the continuous decrease in abundance of adult perch and perch egg strands, hypothesized that the predation pressure exists on a single-level, *i*.*e*. catfish eat spawning perch. According to [33,12], perch is a common part of the catfish diet. Nevertheless, the new finding showing predation on perch egg strands, which were considered to be unpalatable [1,2], throws new light on the predator-prey interaction between catfish and perch.

Since perch (both *P*. *fluviatilis* and *P*. *flavescens*) exhibit a reproductive style unique among teleosts (single ovary is enclosed in a membrane forming an ovisac), the yearly effort of a perch female committed to reproduction is comprised of one item (egg strand) with only one defence–unpalatability [1]. In contrast to pike, catfish seem to be one of a few and presently the only known predators able to digest this easily available food source. The substantial impact on perch reproductive material induced by catfish predation is apparent from the triple decrease in abundance of perch egg strands per adult perch caught after catfish mass stocking in Milada and Most Lakes. From an evolutionary perspective, such behaviour, where catfish utilize the whole reproductive potential of a single perch female, represents a serious bottleneck for gene flow within the perch population. Thus coexistence with catfish presents a serious threat for perch on more than one level.

Catfish utilize egg strands even though they are nutritionally poor due to high water content after expanding in the aquatic environment [1,34]. The advantage is probably the short search and handling time, profitable mainly in cold water during spring time. Therefore, the impact on perch in some localities must be significant as perch egg strands are easily available and may be utilized in high quantity to cover the nutritional needs of catfish. In practice, a significant impact on the perch population is apparent in Milada Lake (Fig 3). A similar trend is also predicted to occur in Most Lake within the following few years.

Considering the overlapping indigenous areas of perch and catfish [35] to the present stage of knowledge the catfish occurrence does not seem to be fatal for perch in these areas. Nevertheless, catfish have been spread to new localities in south-west Europe and south Kazakhstan which may favour this thermophilic species [12]. Any catfish occurrence in a locality with perch has a potential to shrink the gene pool [36] and reduce fitness of at least some perch individual. Evidence of perch egg strands in catfish diet (thus far regarded as unpalatable) demonstrates their generalist behaviour as a top predator of freshwater ecosystems. It shows the extremely wide dietary niche of catfish likewise the study dealing with beaching behaviour of catfish, an impressive method of catching pigeon on beaches [16]. Our finding from Most and Milada Lakes broadens the knowledge about European catfish, its dietary plasticity and impact on lower trophical levels. In addition, we may assume that the spread of catfish to new localities caused by man and by global warming [12, 14] will favour this species at the expense of many other species.

## Supporting Information

S1 Data (XLSX)
